# Health TAPESTRY: co-designing interprofessional primary care programs for older adults using the persona-scenario method

**DOI:** 10.1186/s12875-019-1013-9

**Published:** 2019-09-04

**Authors:** Ruta Valaitis, Jennifer Longaphy, Jenny Ploeg, Gina Agarwal, Doug Oliver, Kalpana Nair, Monika Kastner, Ernie Avilla, Lisa Dolovich

**Affiliations:** 1 School of Nursing, McMaster University, HSC 3N25,1280 Main Street West, Hamilton, Ontario L8S 4K1 Canada; 2 Department of Family Medicine, McMaster University, David Braley Health Sciences Centre, 100 Main Street West, 5th floor, Hamilton, Ontario L8P 1H6 Canada; 3 Institute of Health Policy, Management and Evaluation, University of Toronto, 155 College Street, Toronto, Ontario M5T 3M6 Canada; 4 Department of Medicine, Division of Clinical Immunology & Allergy, HSC 3V47, McMaster University, 1280 Main Street West, Hamilton, Ontario, L8S 4K1 Canada

**Keywords:** Co-production, Co-design, Participatory design, Implementation, Older adult, Primary care, Primary health care, Intervention, Patient engagement, Patient involvement, Interprofessional team, Technology

## Abstract

**Background:**

Working with patients and health care providers to co-design health interventions is gaining global prominence. While co-design of interventions is important for all patients, it is particularly important for older adults who often experience multiple and complex chronic conditions. Persona-scenarios have been used by designers of technology applications. The purpose of this paper is to explore how a modified approach to the persona-scenario method was used to co-design a complex primary health care intervention (Health TAPESTRY) by and for older adults and providers and the value added of this approach.

**Methods:**

The persona-scenario method involved patient and clinician participants from two academically-linked primary care practices. Local prospective volunteers and community service providers (e.g., home care services, support services) were also recruited. Persona-scenario workshops were facilitated by researchers experienced in qualitative methods. Working mostly in homogenous pairs, participants created a fictitious but authentic persona that represented people like themselves. Core components of the Health TAPESTRY intervention were described. Then, participants created a story (scenario) involving their persona and an aspect of the proposed Health TAPESTRY program (e.g., volunteer roles). Two stages of analysis involved descriptive identification of themes, followed by an interpretive phase to extract possible actions and products related to ideas in each theme.

**Results:**

Fourteen persona-scenario workshops were held involving patients (*n* = 15), healthcare providers/community care providers (*n* = 29), community service providers (*n* = 12), and volunteers (*n* = 14). Fifty themes emerged under four Health TAPESTRY components and a fifth category - patient. Eight cross cutting themes highlighted areas integral to the intervention. In total, 414 actions were identified and 406 products were extracted under the themes, of which 44.8% of the products (*n* = 182) were novel. The remaining 224 had been considered by the research team.

**Conclusions:**

The persona-scenario method drew out feasible novel ideas from stakeholders, which expanded on the research team’s original ideas and highlighted interactions among components and stakeholder groups. Many ideas were integrated into the Health TAPESTRY program’s design and implementation. Persona-scenario method added significant value worthy of the added time it required. This method presents a promising alternative to active engagement of multiple stakeholders in the co-design of complex interventions.

**Electronic supplementary material:**

The online version of this article (10.1186/s12875-019-1013-9) contains supplementary material, which is available to authorized users.

## Background

Engaging patients and health care providers to co-design health interventions has gained global prominence [1]. Co-design or co-production is increasingly being used by organizations to ensure that health programs and services are closely aligned to user needs and requirements [2]. Perrott explains that co-design can be viewed as: design for (users as the main input in the design process), design with (users being involved in providing solutions to design problems), and design by (users actively participating in design) [1]. He also describes co-design in health services in the form of experience-based co-design, where emphasis is on designing experiences [3] rather than systems and processes. Perrott [1] argues that it is important to consider relationships that occur between service providers and service users in the design of service interventions.

While co-design of interventions is important for all patient groups [3], it can be argued that it is particularly important for older adults who often experience multiple and complex chronic conditions. An increase in chronic conditions leads to complexity in management and treatment, resulting in challenges in coordinating care and communication across multiple providers [4]. Furthermore, older adults have specific needs related to changing physiologic and psychological abilities that are often ignored in the design and implementation of new products or programs, which can hamper adoption of innovations [5]. While research has determined care preferences of patients with multimorbidity [6], a recent review of interventions for multimorbidity does not address issues of co-design [4, 7].

Given the high prevalence of multimorbidity seen in primary care [8] and the importance interprofessional providers play in supporting older adults with chronic conditions [9], engaging providers in the design of health care interventions is equally important. One of a few papers that addressed this was a co-designed complex intervention (CARE Plus) aimed at adults with multi-morbidity living in socio-economic deprivation [10]. Qualitative focus groups informed the refinement of the intervention taking into account both adults’ and care providers’ perspectives.

Health TAPESTRY (Teams Advancing Patient Experience: Strengthening Quality) is a primary care innovation aimed at promoting optimal aging for Canadians through the integrated use of: *trained community volunteers, technology, interprofessional primary care teams, and community engagement* to support system navigation [11]. This involves a paradigm shift that places the patient at the centre of a broad-based care team (the circle of care), which includes healthcare providers, family members, caregivers, volunteers, members of community organizations having intentional, proactive conversations about a person’s life and health goals as well as health risks. The emerging information is used to create congruent tailored interventions that support achievement of those goals and mitigation of health risks. Health TAPESTRY planning was grounded in the use of developmental evaluation [12] and participatory design methods to engage potential end-users and stakeholders in the development of this complex program. To accomplish this, Health TAPESTRY employed a methodology called persona scenarios which has been used in the e-health field [13]; and was modified for use to co-design a primary health care intervention [14].

Traditional methods for determining program requirements have usually involved focus groups or interviews [15]. The end user becomes a consultant who responds to the researcher’s predefined questions [13]. This places the researcher in the driver’s seat rather than the user of the program or product. The natural tendency for a design team is to be self-centred emphasizing their wants and needs, which can result in the production of products that inadequately perform for the intended users [16]. Since users and designers often have different backgrounds and life experiences, reaching a shared or common understanding of the topic is essential in the development of new programs or products [5]. One solution is the use of persona scenarios. Persona scenarios are created in small workshops where participants work together to create a potential end-user (e.g., a program provider or its recipient) engaged in the program (persona) and a scenario. A scenario is a short story which communicates information from which to draw requirements (what users want the program to do) for an innovation. Persona-scenarios provide added value that cannot be achieved through focus groups. There is a risk in focus groups that designers might project their ideas and mental models into the design and ask for feedback on them. Whereas, persona-scenarios can help to ensure that the design is representative of older adults’ and providers’ experiences in their contexts. Carroll argues that personas provide multiple views of an interaction which can inform developers about consequences of design decisions. [13]

While most often persona scenarios have been developed by designers derived from data gathered from end users [5] such as interviewing users, then synthesizing the interviews and mapping participants, there is some risk that designers project their own ideas, and mental models in the design [17]. Persona scenarios have traditionally been used to design information and communication technologies [13, 18, 19]. In this paper we examine a new approach in the persona-scenario method whereby the design is created by the end users and not by the researchers.

## Methods

This purpose of this paper is to report on how the persona-scenario method was used to co-design a complex primary health care intervention (Health TAPESTRY) by and for older adults and providers and the value added of this approach. The persona-scenario method was used in co-designing the Health TAPESTRY intervention by primary care and community services providers, volunteers as well as the potential recipients of the program. In this way, personas and scenarios could capture all stakeholder’s perspectives. Our study followed the COREQ guidelines.

### Participants

Participants included patients, primary care clinicians, volunteers and community service providers. A convenience sample of patient and clinician participants were recruited from two academically-linked primary care practices in a Family Health Team in an urban setting. The Family Health Team (FHT) model in Ontario, Canada uses interprofessional teams with physicians and other primary health care providers to provide comprehensive services to patients [20]. Efforts were made to include a wide range of older adults participants based on geography, age, and socio-economic status to obtain a variety of ‘stories’ from various contexts. Potential volunteers were recruited from the local university and retired adults. Local community service providers were also recruited who were representative of the anticipated service provider organizations that would provide additional health and social services. [21]. Invitations were sent out by email. Written consent was obtained from all participants.

### Data collection

Details of the persona-scenario method and analysis are described by Valaitis et al. [14]. In brief, persona-scenario workshops were held and facilitated by two female PhD prepared and one female Master’s prepared researchers (RV, KN, JL) highly experienced in qualitative methods and facilitating groups with the help of research assistants. Note takers were also present to assist patient and community provider pairs to take notes to capture their ‘stories’ on a laptop for participants to refer to later. They took place in an accessible university research centre, scheduled at varying times to accommodate participants.

At the beginning of each workshop, facilitators introduced themselves informally to participants and invited them to get some refreshments. When the workshop began, formal introductions were made by the facilitators and participants were informed that the purpose of the workshop was to engage them in co-designing an intervention for a randomized controlled trial. Core components of the Health TAPESTRY intervention were briefly described including: the use of *volunteer* visitors to conduct home visits and transfer information to the clinic using *technology*, and engagement with *community services,* in the context of the *interprofessional primary care team*. While these components framed the study, the facilitators stressed that the intervention itself was still in development and that their input would help to create the details of the intervention and its implementation.

Separate two hour workshops were conducted for patients, primary care clinicians, volunteers and community service providers. Participants were paired with individuals with similar backgrounds. Pairing occurred at the start of the workshop following participants’ introductions. For example, volunteers who reported having significant volunteering experience were paired with other volunteers with a similar amount of experience, while patients were paired based on gender or age or other similar personal characteristic shared during the introductions. Pairing was completed organically and in an informal way with support from the facilitator attempting to create homogenous pairs. In some instances, three people worked together when there was an uneven number of participants attending a workshop.

Working mostly in homogenous pairs, participants were provided with a guide asking them to create a fictitious but authentic persona that represented them or people like themselves (see Additional file 1). They were asked to use their persona in a scenario that represented an aspect of the proposed Health TAPESTRY program (e.g., how an older adult would find out about Health TAPESTRY; the role of the volunteer; how information flows between the patient and physician). Each pair had guiding questions to support story development who were aided by a research facilitator to keep them on track, and record their discussion and final story. Participants were given about 45–60 min to work through the persona and scenario, generating questions and reviewing the captured notes. Once finished, each pair summarized their statements and presented them back in their own voice in the form of a story to the larger group. A large group discussion followed to identify commonalities and differences in the scenarios generated and how they could inform the development of the intervention. The presentations and large group discussion acted as a member check to validate the data. Stories and discussions were digitally recorded and all data were uploaded to NVivo 10 for analysis. Participants were invited to attend a community meeting in the same location where the workshops were held. High level results were shared from the persona-scenarios. Approximately 20–25 participants attended. Although not intended as a formal member check, participants were asked if the results resonated with them based on their input from the workshops providing validation of the results. Overall, participants were very positive about both the experience and the results, which they felt were reflective of their input.

### Analysis

All recordings were transcribed verbatim and checked by a member of the research team for accuracy prior to analysis. As is done in a typical qualitative descriptive approach in analysis [22, 23], transcripts were coded into descriptive qualitative codes staying as close to the data as possible. These codes were then collapsed into themes. For example the code “*Nurse persona becomes the TAPESTRY champion in the FHT, and now feels like he has the tools to make a difference for this famil*y” was collapsed under the theme - *TAPESTRY champion* which was organized under the relevant Health TAPESTRY program component (i.e., *Team*). The coding and development of themes were completed by one researcher (JL) and checked by a second (KN) and the first author (RV). The larger team reviewed this coding structure while engaged in the next phase of analysis to further support rigor. In some cases the same theme emerged in more than one program component. In these cases, the theme was considered cross-cutting and highlighted relationships across components which denoted the complexity in the intervention.

The next phase of analysis was more interpretive. It required defining the activities or actions and products associated with them [17]. Therefore, codes under each theme were interpreted by the full research team in terms of the ACTIONS (what activities or processes need to happen for the event to occur, e.g., *consider who can be the Health TAPESTRY champion within the family health team*) and PRODUCTS (what product/s or items are needed to support the ACTION, e.g., *identify TAPESTRY Champion as system navigator*). Often different actions were required for the same product, or one action required several products to support it. Addressing actions in this way helped to fully define what was needed for implementation.

**Fig. 1:**
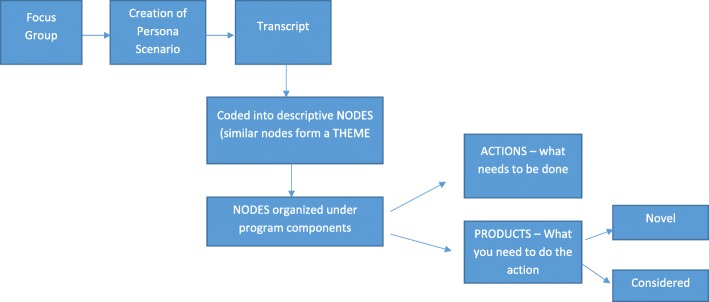
Process for generating actions and products from persona-scenario workshops

We were interested to identify the value added of the persona-scenario method in this project. To address this, we conducted another level of analysis. At least two team members, including research staff and investigators who were heavily involved in designing the intervention, were asked to label all the actions and products under a theme on Excel spreadsheets. They labeled them as either ‘novel’ (ideas that were new to the program team) or ‘considered’ (ideas that were already considered by the team prior to conducting the persona-scenario). This permitted us to quantitatively and qualitatively assess what value the persona-scenario method contributed to the design of the program. In addition, both novel and considered products in each theme were shared with subcommittees charged with developing these aspects of the project for discussion and consideration in development and implementation of the components of the intervention. This delineation was helpful for understanding how the perspectives of participants influenced the design of the intervention. All steps of the analysis were a collaborative process between research staff and study investigators, and decisions were made through discussion and consensus. See Fig. 1 for a schematic of the analytic process and Table 1 for two examples.

**Table 1:** Examples of actions and products derived from qualitative codes and how these were used by the team

Data Analysis Process	Example 1	Example 2
Code (Quote from group)	“Volunteers are doing more things like training on community literacy or friendly visiting or perhaps providing transportation for the patients” *Community Clinicians Group 2*	“(Nurse persona) becomes the TAPESTRY champion in the Family Health Team, and now feels like he has the tools to make a difference for this family.” *Primary Care Group 1*
Health TAPESTRY Component	Volunteers	Interprofessional Primary Care Team
Theme	Procedures for volunteer visits	TAPESTRY Champion
Action	Create policies and procedures for volunteers	Consider who can be the Health TAPESTRY champion within the family health team
Product	Policy outlining volunteer assisting patients with transportation	Identified TAPESTRY Champion
Novel/Considered	Had already been considered	Novel
How information used by team	Incorporated policy into volunteer training and reference manual	Discussion with clinical team about having a point person to facilitate Health TAPESTRY

## Results

In the spring of 2015, 14 persona-scenario workshops were held over 5 months with 70 participants: patients (*n* = 15), healthcare providers/community care providers (*n* = 29), community service providers (*n* = 12), and volunteers (*n* = 14). Healthcare providers included family physicians both in group and solo practices, nurse practitioners, clinical pharmacists, occupational therapists, and patient navigators (among others). Community service providers included representatives from community organizations such as the YMCA, Heart and Stroke Foundation, Canadian National Institute for the Blind, community pharmacies that hosted blood pressure monitoring, Meals on Wheels and others offering services to older adults in the community. Volunteers tended to be university students in undergraduate programs at the local university, along with volunteers who were often retired teachers, nurses and other professionals recruited from organizations such as a home care service agency. They represented people with backgrounds that were envisaged as being eligible and suited to become Health TAPESTRY volunteers. Workshops ranged from three to eight participants. Two to four researchers were also present, one who facilitated the exercise and others to keep participants on track and take notes for them so that they could focus on creating their personas and scenarios. Overall, 33 persona-scenarios were generated representing authentic but fictitious Health TAPESTRY participants including volunteers, clinicians, community service providers and patients. (Additional file 1 presents examples of personas).

Clinician groups brought knowledge and experiences related to needs of older adults, clinic and team functioning, and potential enablers and barriers to clinic efficiency. Patients brought perspectives related to their personal needs, strengths, concerns, and limitations. Community agency participants reminded the team of the need for inter-agency community and collaboration, as well as highlighting fiscal realities of smaller community agencies that depend on referrals from others, such as primary care, for their funding. Prospective Health TAPESTRY volunteers brought perspectives related to roles that they could play (e.g., data collection, supporting older adults, linking to community agencies) as well as content for their training.

Themes were organized in columns under the Health TAPESTRY components (i.e., interprofessional primary care team, volunteers, community services, technology), in addition to a fifth category - patient (Table 2). Table 3 highlights eight additional cross cutting themes which played an important role in the development of the intervention as they drew attention to areas that were integral to its development and success. Cross-cutting themes included: (a) team use of information, (b) role clarity, (c) logistics, (d) privacy and confidentiality, (e) using the personal health record, (f) communication, (g) enrolment and publicity, and (h) connection. Table 4 illustrates how a cross-cutting theme (i.e., role clarity) informed multiple components of the intervention. It also demonstrates how products under this theme were implemented or deemed to be not feasible.

**Table 2:** Themes for intervention components and patient

Inter-professional Primary Care Team	Patients	Volunteers	Community Services	Technology
1. System Navigation	1. Family	1. Rapport and trust	1. Designated roles	1. Patient/Family Guide to the PHR
2. TAPESTRY champion	2. Medications	2. Volunteer satisfaction	2. Privacy and consent	2. Medication management
3. Designated Roles	3. Home visiting	3. Tasks performed by volunteers/ information gathering	3. Care Coordination	3. EMR requirements
4. Enrolment into program	4. Enrolment	4. Publicity and community communication plan	4. Information sharing	4. Record accessibility
5. Triaging	5. Care planning	5. Volunteer training		5. PHR to monitor patient over time
6. Standard operating procedure	6. Tracking	6. Procedures for volunteer visits		6. Validation and data checking
7. Referrals	7. PHR	7. Volunteer helping patient with PHR		7. Technology failure
8. Follow up on volunteer Information	8. Consent/ privacy/ confidentiality	8. Electronic communication plan		8. Personal Health Record- TAPESTRY App generated reports
9. Scheduling	9. Communication	9. Scheduling		9. Volunteer Using PHR
10. Information sharing across PC team	10. Patient Volunteer relations	10. Communications between Volunteer and Clinic		10. EMR facilitates communication to community services
	11. Finances	11. Volunteer coordinator		11. Tablet costs
		12. Volunteer matching (pairs)		12. Flagged entry of TAPESTRY information
				13. Communication with team

**Table 3:** Cross-cutting themes that emerged from the persona scenario groups

Cross-cutting theme	Definition	Component
Team use of information	The collection of information and generation of reports sent to the clinical team including: - flagged information - how information is distilled into a report - how report is received and initially assessed - follow up upon including referrals	Interprofessional Primary Care Team
		Technology
Role clarity	Defined tasks performed by clinicians, volunteers, and community members including: - specific new roles (e.g., volunteer coordinator and TAPESTRY champion) - general interactions between volunteers, patients and their families - expectations given during volunteer training.	Interprofessional Primary Care Team
		Volunteers
		Community Services
		Patients
Logistics	Program information about: - managing team coordination - volunteer matching - scheduling home visits - ensuring feasibility of IT components	Interprofessional Primary Care Team
		Volunteers
		Technology
		Patients
Privacy and Confidentiality	Related to: - communicating and information sharing in a safe and protected way, between all actors (facilitated with technology) - building trust - gaining consent from patients - ensuring data accuracy and accessibility agreements.	Volunteers
		Community Services
		Technology
		Patients
Using the personal health record	Related to the use of the PHR: - by patients, with or without the assistance of volunteers - the volunteer to facilitate communication - the team to monitor patients	Technology
		Patients
Communication	Relaying information: - between patients (via the volunteer) and the clinic - considering how the team and volunteers would communicate their experiences	Volunteers
		Technology
		Patients
Enrollment and publicity	Recruitment and enrollment of clinical teams and individual patients in Health TAPESTRY including: - the overall communication plan - public face (TAPESTRY marketing campaign)	Interprofessional Primary Care Team
		Volunteers
		Patients
Connection	Coordination of care to patients including: - within the clinic - out to the community - facilitated by system navigation	Interprofessional Primary Care Team
		Community Services
		Patients
		Technology

**Table 4:** Example of a cross cutting theme - Role Clarity - informing intervention design

Role Clarity				
Component	Theme	Sample Product^a^	Quote	How implemented
Interprofessional Primary Health Care Team	Health TAPESTRY champion	Identifies Health TAPESTRY Champion to act as system navigator (novel)	“(Nurse persona) becomes the TAPESTRY champion in the family health team, and now feels like he has the tools to make a difference for this family.” *Primary Care Group 1*	Discussion with clinical team about having point person to facilitate TAPESTRY
Volunteers	Procedures for volunteer visits	Policy outlining volunteer assisting patients with transportation (considered)	“(We) envision the volunteers doing more things like training on community literacy or friendly visiting or perhaps providing transportation for the patients” *Community Clinician Group 2* “Maybe the volunteer could put them in contact with some other services like the DARTS (*disabled and aged regional transportation system*) bus or some sort of driving shuttle.” *Volunteer Group 1*	Incorporated policy into volunteer training and reference manual
Patient	Patient/Volunteer relations	Volunteer photo provided to patient in advance (novel)	“So we were worrying about the idea of scammers and things like that. So we said that the volunteer should be showing photo ID at the door, like through the peep hole or what not, before the senior actually lets them into the house” *Patient Group 3* “And the volunteer that she knows is there to essentially to provide a sense of security. So TAPESTRY should be providing a picture of the volunteer who she doesn’t know to the patient prior to the volunteer showing up that way the patient can put it up on their fridge and kind of act as a reminder. And if they have dementia or something, it’s easy for them to see the picture and just quickly compare as opposed to having to remember what their face looks like. ”*Patient Group 2*	Clear identification, notification and security during volunteer visits.
Inter-professional Primary Health Care Team	Designated roles	List of users for each community agency (Novel)	“They could look for names of their clients, because again what we have seen in the community is that there are many times where it looks like the client does not have anybody involved, but when you actually start getting involved and/or making referrals, you find out people are actually hooked up to a number of services that, that don’t always, provide that information.” *Community Service Provider Group 2, July 4, 2013*	Determined not feasible to implement

^a^Please note that for each example, only one item was shown to illustrate the process rather than providing an entire list of concepts

Table 2 provides examples of how information gathered from persona-scenarios was used in the development of the Health TAPESTRY intervention. In total 414 actions and 406 products were extracted from all themes. Of the 406 products, almost 44.8% (*n* = 182) were considered to be novel, while 224 had already been taken into account by the research team. The final list of actions and products organized under Health TAPESTRY components and themes was circulated widely to study leads and program staff. This information was discussed at various stages of implementation such as the development of the volunteer program and volunteer recruitment, protocol writing, ethics applications, patient recruitment and communications to the clinic.

### Outcomes: consideration of the difference between participant data and researcher plans

The 182 novel product ideas were identified across all themes and intervention components. Many of the items were considered novel and feasible and were integrated in some way in the intervention. Under the *volunteer* component, selected novel ideas included: training issues (e.g., volunteer training by clinicians to improve trust; training related to behaviour issues, first aid, and health literacy). For the *interprofessional primary care team* component, examples of novel ideas included: marketing and enrolment products for Health TAPESTRY clinicians; introduction of a Health TAPESTRY champion on the team; standard operating procedures (e.g., follow up based on information from volunteers, and linking patients to interprofessional team members such as social workers). In relation to the *community* component, there were many novel ideas such as: case conference discussion process that includes community agency considerations; a formal announcement to community partners of the program launch; the development of a list of community resources to which the team was comfortable to make referrals. With respect to the *technology* component, many of the novel ideas raised were not feasible due to the reasons noted earlier. However a number of them were applied [e.g., the electronic medical record (EMR) monitors information about patient’s status over time; the Health TAPESTRY app has places to indicate progress and observations from the volunteer for the family doctor to monitor; and, there is a notification system from the Health TAPESTRY app to the volunteer coordinator]. With respect to issues related to *patients*, there were many novel ideas raised that incorporated products to support the family in the Health TAPESTRY intervention such as: a calendar of volunteer visits for the family; a caregiver burden assessment; and system navigation services accessible to patients and their families.

Sometimes actions and products generated from the persona-scenarios were not practical, feasible, nor judged to be acceptable due to inadequate resources (a computerized matching system to pair volunteers; a process for a community services worker to schedule a primary care visit; a forum for patients and patients' families to discuss difficulties); and, other barriers such as privacy laws (e.g., community health care worker access to the Health TAPESTRY care plan within the primary care Electronic Health Record (EMR); community service provider messaging option between agencies; EMR communication between community organizations and the clinician; a searchable list of Health TAPESRTY patients for community services to improve communication). Products such as these created a point of tension between the needs of group participants and what was feasible and pragmatic. In this case, the team identified that it was ultimately the spirit of cross communication that was being highlighted, and in future Health TAPESTRY activities these issues would be explored more fully. Some ideas were difficult to integrate in the short term due to the stage of the intervention’s evaluation, such as, an engagement strategy to increase clinician uptake of Health TAPESTRY using evidence to show value of the program/value added, and a provincial funding model for participating in Health TAPESTRY for all clinicians to join.

During persona scenario workshops, participants were asked to identify any barriers they foresaw in the Health TAPESTRY program implmentation. Some of the actions generated from these suggestions directly contradicted implementation plans considered by the Health TAPESTRY team. For example, the research team had initially suggested having volunteers use tablets to take photos of a patient or their home as a means of reporting back information to the health care team. However, when patients considered this, issues of privacy and concerns about security made it an unviable option. Hearing the voice of patients was critical for discerning feasibility of various aspects of the proposed intervention.

Other tensions arose on two occasions when participants in workshops rejected the Health TAPESTRY approach. They used their persona scenario discussions to outline how they felt that the program would not be able to help patients or would burden an already-busy community clinician. While these scenarios did not yield many products to inform the intervention, they became meaningful as they represented real concerns and challenges the Health TAPESTRY team would need to face to demonstrate the value of the program, foster uptake and ensure sustainability. Scenarios detailing clinician persona’s concerns (time constraints, burden, and lack of trust in the role of the volunteer) and highlighting areas of patient need (mental health, optimal aging) impacted communications within the project and understanding of the project’s context.

## Discussion

Overall, the persona scenario methodology was well received by participants. This method was flexible in identifying themes specific to different components of the intervention and in identifying cross cutting themes that applied to multiple components including themes that integrated components. Results also showed that there was value added, as almost half of the ideas emerging were in the form of new ideas not previously considered by the research team that informed the final design of all components of the Health TAPESTRY intervention as well as its implementation. The research team benefited from information gathered that was specific to each participant group (i.e., patients, volunteers, primary care and community service providers). Each group brought their lived experiences which helped shape their stories. Such insights helped to define details of the intervention and implementation that addressed all stakeholders’ perspectives.

As we experienced, a benefit of persona-scenarios was that all stakeholder groups were asked to think about interactions with all other groups, which informed design decisions [13]. This resulted in gaining a deep understanding about the relationships among stakeholder groups and among components of the intervention. Sharing perceptions with all stakeholder groups can highlight potential conflicts early in the development process. This can help ensure that the intervention and its implementation will be more acceptable, relevant and feasible from each stakeholder groups’ perspective.

Additionally, developing detailed specifications of an intervention has the potential to assist others in scaling up. The World Health Organization’s Expandnet Framework for scale up identifies systems thinking as one of four principles that underpins the process. Systems thinking refers to increasing awareness of the “interrelationships between the innovation, the user organization, the resource team and the larger environment within which scaling up takes place.” [24] (Page 8) Although Health TAPESTRY was not yet ready for scale up at the time of this study, persona-scenario results provided information about such interrelationships through broad stakeholder participation. This included clinical teams in primary care organizations, community service agencies, experienced volunteers, as well as patients.

Empirical research has been criticised for focusing only on “short-term adoption of simple innovations by individual adopters” (p. e367) and non-adoption of innovations, while studies exploring local adaptations, scale and spread have been rare [25]. Having clear detailed descriptions of intervention core components allows for the evaluation of core functions of the intervention and can help to make better decisions about non-core components that can be adapted to new contexts [26]. A recent systematic review of effective strategies for scaling up evidence-based practices in primary care indicated that scale up strategies were poorly described and provided little measurable evidence regarding success [27]. Future research is encouraged to explore if there is a place for the persona-scenario method to fill this gap and to help determine where and if adaptations of tested and effective complex interventions are needed during scale up.

Health care program design using persona scenarios is inherently different than other methods commonly used for program co-design. Persona-scenarios are typically developed by a design team (i.e., the researchers) based on data gathered from sources such as focus groups, interviews and/or field observations to gain an understanding of end users’ contexts and processes used in usual practice [5, 17, 18]. Researchers thereby are filtering the information from the data that they collect. Whereas, in the persona-scenario method described here, end users developed the personas and scenarios and the potential for misinterpretation of the data is reduced. This approach also allowed us to create multiple personas from numerous sources uninhibited by pre-set program design, other than a high level knowledge of the program’s core components. Additionally, group discussions have been used to collect feedback following a pilot study to inform required adjustments before rolling out a full trial [10]. Persona-scenarios make space for the creativity and thoughtful reflection prior to the implementation of a pilot or trial, with participants drawing from their personal and professional experiences and desires for improvement. Utilizing this approach allowed the intervention design to more fully capture each type of perspective and anticipate potential implementation challenges. In essence, the persona scenario method can be considered a knowledge translation and exchange intervention that informs the development of an intervention involving its end users as well as those delivering the intervention.

Though the persona group method has been shown to have significant added value, there are some challenges and limitations to its use in the primary care program development arena. The process of participant recruitment is time consuming especially in relation to health care providers. Flexibility is critical as employed patients are more likely to be available in the evenings while retired older patients are more likely to be available in the afternoons. In addition, incentives will be required to encourage attendance (e.g., costs to cover parking, food, as well as transportation assistance) particularly for patients and volunteers. It is important that facilitators have familiarity with qualitative methods and are skilled in leading group discussions and probing for rich data. This is particularly important if participants push back on the planned components of the intervention as was mentioned above. Without careful facilitation and management of the process, negativity can quickly erode contributions of the entire group. Furthermore, the large volume of information produced was difficult to disseminate to scientific leads and was unwieldy to review in team meetings. This was partially resolved by organizing materials by theme so that smaller sections of data could be reviewed in smaller chunks. Another challenge was the time required to perform the analysis. Future use of this method, should build in extra time for coding and data analysis. While the facilitation and analysis of the data is resource intensive [14], participants enjoyed the process and provided positive feedback overall. The persona scenario methodology was an effective and useful way to engage stakeholders and get rich descriptions of the intervention and its implementation despite the challenges in the approach.

### Study strengths and limitations

Persona-scenarios participants need to be representative of the stakeholder group/s to be receiving or delivering the intervention. Greenhalgh and colleagues used narrative story-telling to inform the design of culturally-congruent diabetes self-management program by minority cultural groups [28]. After analysing 300 informal stories of people living with diabetes, they found many commonalities among the storylines. This begs the question of how many people and how many stories are enough? The concept of “saturation” could be a useful guide. A principle of data saturation in qualitative research is that there is no new data, themes, or coding and it is possible to replicate the study, however determining how to reach saturation will vary by study design [29]. In our study, we gathered information on 50 themes with 33 persona-scenarios. The themes acting as a useful organizing framework, with the codes providing details needed to determine design specifications under these themes. The codes were very descriptive staying close to the data which provided richness in results. Most ideas (codes) were only identified once or repeated a few times indicating that the concept of saturation may not be appropriate for the persona-scenario method. In qualitative interviews, Baker and Edwards [30] argue that it is difficult to determine sample size in advance since saturation should be used as the guide to how many cases are enough. However, they also argue that the depth of the data needs also to be considered: How rich (quality) and thick (quantity) is the data? We would argue that the quality and quantity of ideas and the novel ideas that were identified were significant and illustrate the value added and strength of this study. Available resources and time determined when we had to stop collecting data.

In addition, it is important when co-designing an intervention that participants involved are representative of the population to be served and of those who will deliver the service. We directly engaged providers in the organization who would deliver the intervention and prospective volunteers from the organizations where we planned to recruit them. However, we could have recruited a larger number of older adults from more diverse backgrounds (i.e., ethnicity, income, and literacy levels). Another consideration is transferability of results. As in other qualitative studies involving data collection methods such as focus groups or interviews, transferability of results is up to the reader to determine based on similarities in context between the study populations and contexts. This points holds true for the persona-scenario method.

## Conclusion

The persona-scenario method resulted in the creation of over 400 novel ideas, many of which were integrated into the Health TAPESTRY program’s design as well as its implementation. These ideas went well beyond what the research team had originally intended illustrating a significant added value worthy of the added time it required. The persona-scenario method in which stakeholders were the designers provided rich descriptions of the components and interactions between them, as well as the implementation processes of a complex primary care intervention. Another important added benefit of persona-scenarios was that stakeholder groups considered interactions with all other groups. Results reflected each stakeholder groups’ perspectives taking into account the unique context of each group as well as the relationships between the stakeholder groups and across the components of the intervention. Although co-design by end-users of an intervention using the persona-scenario method requires further study to fully explore its benefits and drawbacks and most effective strategies, the method adds a promising alternative to the co-design of complex primary health care interventions that actively engages multiple stakeholders including patients and providers.

## Additional file

Additional file 1: Persona Examples. This file provides a table containing examples of characteristics of personas developed by participants representing a clinician, volunteer, community service provider and patient. (DOCX 16 kb)

## Data Availability

The dataset generated and/or analysed during the current study are not publicly available since we did not obtain consent for this from participants and releasing the raw data could compromise participant confidentiality.

## Abbreviations

EMR: Electronic Medical Record
FHT: Family Health Team
