# Supplementary material for: Health TAPESTRY: co-designing interprofessional primary care programs for older adults using the persona-scenario method
Source: BMC Fam Pract. 2019 Sep 4;20:122. doi: 10.1186/s12875-019-1013-9 (PMC6727539; doi:10.1186/s12875-019-1013-9)
Supplement: Supplementary file 1 — Persona Examples. This file provides a table containing examples of characteristics of personas developed by participants representing a clinician, volunteer, community service provider and patient. (DOCX 16 kb) [file 12875_2019_1013_MOESM1_ESM.docx]

Additional file 1: Persona Examples

| **Persona** | | | | |
| --- | --- | --- | --- | --- |
| **Type of Persona** | **Clinician** | **Volunteer** | **Community Service Provider** | **Patient** |
| **Name** | Brenda | Andrew | Ms. Hamilton | Susan |
| **Discipline** | Occupational Therapist | Undergrad Student - Sciences | Manager of Supportive Housing for older adults | Teacher |
| **Age** | 35 | 21 | 40 | 70 |
| **Gender** | Female | Male | Female | Female |
| **Desires** | Continuity of care | To be working with people on a daily basis |  | Health conscious – Aims to stay healthy and stay alive as long as possible. Critical of the health system. Caregivers have incomplete education. |
| **Attitudes Re: Work** | Passionate about her work. She's optimistic. Wants a more established relationship with her patients. | Passionate about the elderly, good active listener. | Passionate about seniors, community minded. |  |
| **Education** | Masters of Occupational Therapy | High school diploma. Currently completing undergrad in Sciences | McMaster University, B.A. in Social Work | BA, Math degree |
| **Employment Background** | Most of her history is working in a Toronto hospital setting. Left the hospital setting to come to FHT for a longer term better continuity of care with patients. She was looking for a more established relationship. Now a part of Niagara FHT for past 2 years. | Part time employment at McDonalds for the past 3 years. | Previously worked part time in a group home and a community support centre. Currently a manager of Supportive Housing for older adults. | Retired teacher |
| **Organizational Characteristics** | Family Health Team |  |  | n/a |
| **Type/Size of Community** | Niagara. Medium sized urban centre | Large metropolitan centre | Large urban centre. | Urban Centre |
| **Years of Service with Organization** | 2 years | 3 years | 10 years | n/a |
| **Experience with Volunteers/ing** | Experience with volunteers mostly in hospital setting where she coordinated volunteers and identified patients that required a volunteer in the hospital (ex: patients with brain injury etc.) | 4 years of volunteering experience in a hospital and at a nursing home as an activity leader. | Lots of experience with volunteers. | Cared for family member until death. Some unsuccessful experiences with volunteers. |
| **Experience with Family Health Team Members** | Interacted with a variety of disciplines in the hospital but not working as a team as much as she is now in primary care. Past 2 years has gained a lot of experience in the team approach. Most of her experience was with acute care. Now she is getting more experience with primary care and home care over longer spans of time | Worked with nurses in the hospital where he volunteered. Otherwise no experience. | Lots of experience with primary health care team members, advocacy, helping other navigate the system |  |
| **Experience with Community Agencies** | Very little experience working with community agencies other than Community Care Access Centre. She belongs to the "Y" as well. | Worked with fellow classmates in a community organization. Experience with the YMCA where he volunteered as homework tutor. |  | Experienced with adult day programs for seniors, home care and transportation services for disabled. |
| **Comfort/Experience with Technology** | Very comfortable with technology | Very comfortable with technology, uses laptop and smartphone regularly to enhance learning process | Moderately comfortable with technology however is not seeking new technology | Owns and is comfortable with computers. Uses computer for everything: to look up research information on the web. |
| **Hopes & Fears about Healthcare System** | Wants to see patients and clients as successful as possible in their homes or in the community. Working in primary care has made her more optimistic about her work. She is concerned that the system won't catch up to the needs and the volume, particularly with the aging population. | Hopes to enhance the condition of seniors. Fears not being able to increase the quality of life of seniors and not being able to reach them. | Worried about lack of resources in the system and coping with increasing needs of the clients. Also worried about lack of funding. She feels isolated within the system. |  |
| **Current Health Status** |  |  |  | Has multiple illnesses. Recently widowed. |
